# Annexin A5 Maintains Mitochondrial Integrity by Inhibiting mPTP Opening to Protect Against Acetaminophen-Induced Acute Liver Injury

**DOI:** 10.3390/ijms27177771

**Published:** 2026-08-30

**Authors:** Xiaowen Zhang, Wenwei Li, Luqi Li, Ying Wang, Wei Tang, Jing Zhang, Zichun Hua

**Affiliations:** 1 The State Key Laboratory of Pharmaceutical Biotechnology, School of Life Sciences, Nanjing University, Nanjing 210023, China; 2 Changzhou High-Tech Research Institute of Nanjing University, Changzhou 213164, China; 3 Faculty of Pharmaceutical Sciences, Xinxiang Medical University, Xinxiang 453002, China

**Keywords:** liver injury, APAP toxicity, AnxA5, mitochondria, mPTP opening

## Abstract

Acetaminophen (APAP) represents a major cause of drug-induced liver injury (DILI), and effective pharmacological interventions remain limited. Annexin A5 (AnxA5), a Ca^2+^-dependent phospholipid-binding protein, participates in diverse biological processes related to tissue repair. In this study, we evaluated AnxA5 expression in APAP-challenged mouse livers and clinical samples from patients with liver injury. Using hepatic cell lines AML12 and HepG2, we performed overexpression-based functional assays to assess the cytoprotective effects of AnxA5 against APAP toxicity. Co-immunoprecipitation assays were applied to characterize protein interactions, and mitochondrial functional parameters were measured to dissect the underlying molecular mechanism. We found that AnxA5 was significantly upregulated in both APAP exposed mice and APAP DILI patients. Cellular functional assays showed that AnxA5 overexpression mitigated APAP triggered cytotoxicity in AML12 and HepG2 cells. Mechanistically, AnxA5 bound to voltage dependent anion channel 1 (VDAC1), restrained VDAC1 mediated mitochondrial Ca^2+^ influx, and suppressed VDAC1 oligomerization, which further inhibited mitochondrial permeability transition pore (mPTP) opening. In an APAP-induced liver injury mouse model, exogenous recombinant AnxA5 treatment maintained mitochondrial integrity and ameliorated hepatic inflammation and liver damage. Collectively, our data reveal AnxA5 as an endogenous mitochondrial protective factor and support its therapeutic potential against APAP-induced liver injury.

## 1. Introduction

The liver is a vital organ responsible for essential physiological functions, including metabolism, synthesis, and detoxification. A wide range of insults, such as viral infection, drug toxicity and excessive alcohol consumption, can trigger acute liver injury or contribute to chronic liver diseases [1]. Drug-induced liver injury (DILI) is a particularly common form of acute liver damage and represents the leading cause of acute liver failure (ALF), posing a major clinical challenge [2]. Acetaminophen (APAP), a widely used analgesic and antipyretic, is safe at therapeutic doses but can cause severe hepatotoxicity upon overdose, representing the most frequent etiology of ALF in many countries [3,4]. Beyond APAP, numerous other drugs, including the antifungal ketoconazole, the antiarrhythmic amiodarone, and the antitumor agent methotrexate, have been shown to induce liver injury by impairing mitochondrial function [5,6]. Despite the clinical importance of DILI, effective therapeutic options remain limited, underscoring the urgent need to identify novel protective factors.

Mitochondrial dysfunction is recognized as a central event in the pathogenesis of various liver diseases, including DILI [7]. For instance, APAP overdose generates the toxic metabolite N-acetyl-p-benzoquinone imine (NAPQI), which depletes glutathione (GSH) and triggers mitochondrial oxidative stress [8]. The subsequent opening of the mitochondrial permeability transition pore (mPTP) serves as a critical executioner of hepatocyte necrosis, and its opening is governed by upstream stressors, including oxidative stress and mitochondrial Ca^2+^ overload, both of which are prominent features of APAP hepatotoxicity [9]. Given the central role of mitochondria in DILI, the exploration of mechanisms and strategies for protecting mitochondrial integrity are of considerable therapeutic significance.

Annexin A5 (AnxA5) is a Ca^2+^-dependent phospholipid-binding protein widely expressed throughout the body. In resting cells, AnxA5 is predominantly localized in the cytoplasm. Upon intracellular Ca^2^ elevation, AnxA5 associates with various subcellular compartments in a Ca^2+^-dependent manner, including mitochondria [10,11]. Accumulating evidence has implicated ANXA5 in the regulation of mitochondrial function and cell death. Notably, AnxA5 interacts with voltage-dependent anion channel 1 (VDAC1) to control mitochondrial Ca^2+^ homeostasis, thereby influencing cell fate in response to apoptotic stimuli [12]. Conversely, AnxA5 depletion has been reported to enhance resistance to Ca^2+^-induced apoptosis triggered by staurosporine, H_2_O_2_, or cisplatin [13,14]. Moreover, preclinical studies demonstrate that AnxA5 confers protection across multiple disease models, including ischemic stroke, myocardial ischemia–reperfusion injury, and sepsis [15,16,17]. However, the functional role of AnxA5 in drug-induced liver injury remains unexplored, and its precise impact on mitochondrial homeostasis also needs further investigation.

In this study, we demonstrate that AnxA5 confers protection against APAP-induced acute liver injury both in vitro and in vivo. Mechanistically, AnxA5 prevents VDAC1-mediated mPTP opening. Importantly, treatment with recombinant AnxA5 protein effectively alleviates APAP-induced oxidative stress and mitochondrial damage. Our findings identify ANXA5 as an endogenous protective factor and position it as a promising therapeutic candidate for drug-induced liver injury.

## 2. Results

### 2.1. Upregulation of AnxA5 in APAP-Induced Liver Injury

**Figure 1:**
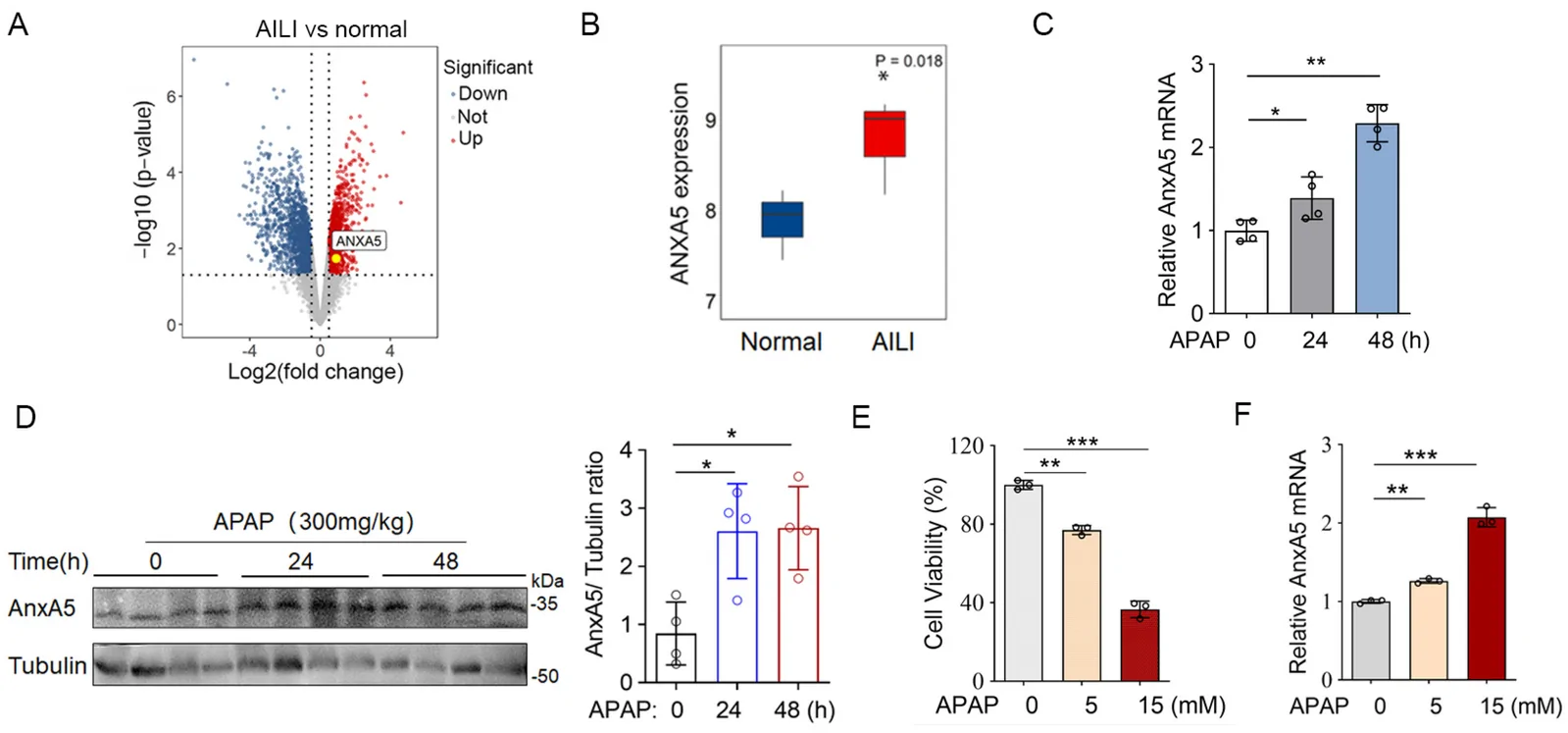
AnxA5 is upregulated in response to APAP-induced liver injury. (**A**) AnxA5 marked in volcano plot of the differentially expressed genes between healthy individuals (Normal) and patients with APAP-induced liver injury (AILI) (*n* = 3). The vertical dashed lines mark the fold-change cutoff at 1.5-fold (|Log2FC| = 0.58), while the horizontal dashed line denotes the statistical significance threshold of *p* = 0.05 (−log10*p* = 1.30). Genes falling outside these boundaries were classified as differentially expressed. (**B**) Relative AnxA5 mRNA expression level in normal and injury liver (*n* = 3). (**C**) QPCR analysis of hepatic AnxA5 mRNA levels in APAP-challenged mice (*n* = 4 each group). (**D**) Immunoblot analysis of AnxA5 protein level in livers from APAP model mice (*n* = 4 each group). (**E**) Cell viability measured by CCK-8 assay in HepG2 cells treated with APAP for 24 h. (**F**) Relative AnxA5 mRNA levels determined by qPCR in the same cell samples from (**E**). Data presented as mean ± SD. * *p* < 0.05, ** *p* < 0.01, *** *p* < 0.001.

To explore the correlation of AnxA5 expression with liver injury, we first analyzed a clinical transcriptomic dataset (GEO: GSE120652), containing gene expression profiles from patients with acetaminophen-induced liver injury (AILI) [18]. Compared with healthy controls, *AnxA5* expression was significantly upregulated in AILI patients (*p* < 0.05) (Figure 1A,B). To validate this clinical finding, we examined ANXA5 expression in a mouse model of APAP-induced liver injury. Compared with the normal reddish-brown appearance of control livers, APAP-treated livers exhibited a yellowish discoloration, accompanied by markedly elevated serum AST levels (Figure S1). In liver tissue, both ANXA5 protein and mRNA levels were significantly increased following APAP challenge (Figure 1C,D). Consistent with these in vivo observations, exposure of HepG2 cells to APAP resulted in a significant decrease in cell viability and a concomitant increase in ANXA5 expression (Figure 1E,F). Collectively, these results demonstrate that ANXA5 upregulation is closely associated with APAP-induced liver injury, suggesting its potential role as a stress-responsive factor in hepatotoxicity.

### 2.2. Protective Role of AnxA5 Against APAP-Induced Toxicity

**Figure 2:**
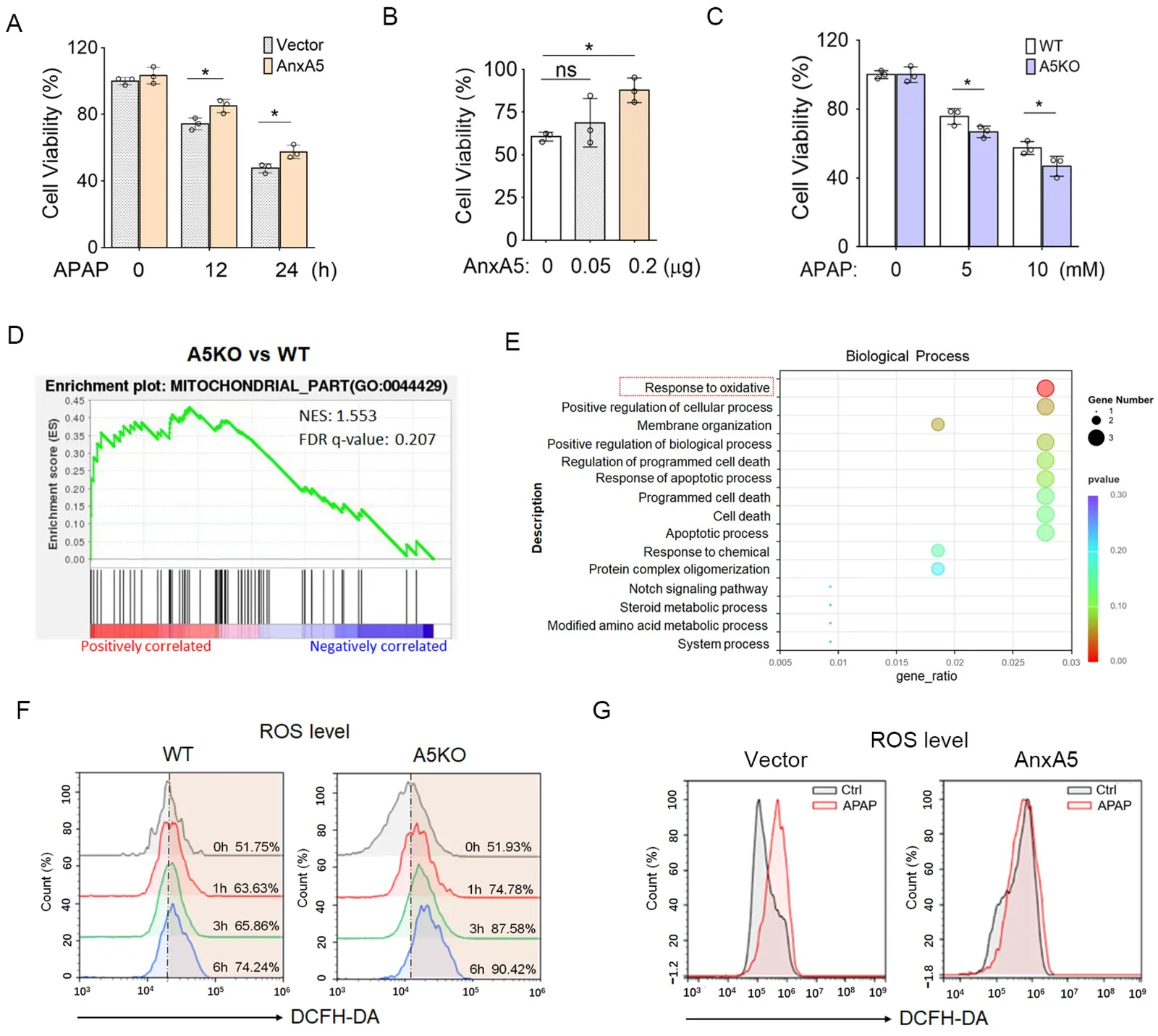
AnxA5 alleviates APAP induced cytotoxicity by reducing mitochondrial stress. (**A**) Cell viability was assessed by CCK-8 assay in HepG2 cells transfected with AnxA5 or empty vector and treated with 10 mM APAP for 0–24 h. (**B**) After transfection with AnxA5 for 24 h, HepG2 cells were treated with 10 mM APAP for an additional 24 h and then subjected to CCK-8 assays. (**C**) Cell viability of wild-type (WT) and AnxA5-knockout (A5KO) HepG2 cells treated with 0–10 mM APAP for 24 h. (**D**) GSEA showing a significant upregulation of mitochondrial-related gene sets in A5KO cells (NES > 1, FDR q-value < 0.25). (**E**) Go enrichment in the Biological Process (BP) category. (**F**) Flow cytometric analysis of intracellular ROS levels using DCFH-DA staining in WT and A5KO cells treated with 5 µM rotenone for the indicated durations. (**G**) Representative flow cytometric analysis of ROS level in HepG2 transfected with AnxA5 or empty vector, with or without 10 mM APAP treatment. Data are presented as mean ± SD. ns: *p* > 0.05, * *p* < 0.05.

To investigate the functional role of AnxA5 in drug-induced hepatotoxicity, we overexpressed AnxA5 in HepG2 cells prior to APAP exposure. AnxA5 overexpression enhanced cell viability in both time- and dose-dependent manners (Figure 2A,B). To further assess the function of AnxA5, we generated AnxA5-knockout (A5KO) HepG2 cells using CRISPR/Cas9 (Figure S2A). Consistent with the overexpression results, A5KO cells exhibited increased susceptibility to APAP-induced toxicity compared to wild-type (WT) cells (Figure 2C). To gain mechanistic insight, we performed RNA-seq analysis on A5KO and WT HepG2 cells (Figure S2B). Gene set enrichment analysis (GSEA) revealed a significant upregulation of mitochondrial-related pathways in A5KO cells (Figure 2D). In line with enrichment analysis, electron microscopy confirmed substantial alterations in mitochondrial morphology in A5KO cells, including a significant increase in mitochondrial length and area (Figure S2C). Gene Ontology (GO) enrichment analysis further linked AnxA5 to biological processes associated with oxidative stress (Figure 2E). To validate it, we used rotenone (a specific inhibitor of mitochondrial complex I) to induce mitochondrial oxidative stress and found that A5KO cells displayed higher levels of ROS compared with WT cells (Figure 2F). Consistently, overexpression of AnxA5 effectively reduced APAP-induced ROS accumulation (Figure 2G). Central to the pathogenesis of APAP-induced liver injury is mitochondrial dysfunction, our data suggest that AnxA5 serves a protective role against drug-induced mitochondrial dysregulation.

### 2.3. AnxA5 Inhibits VDAC1-Mediated mPTP Opening

**Figure 3:**
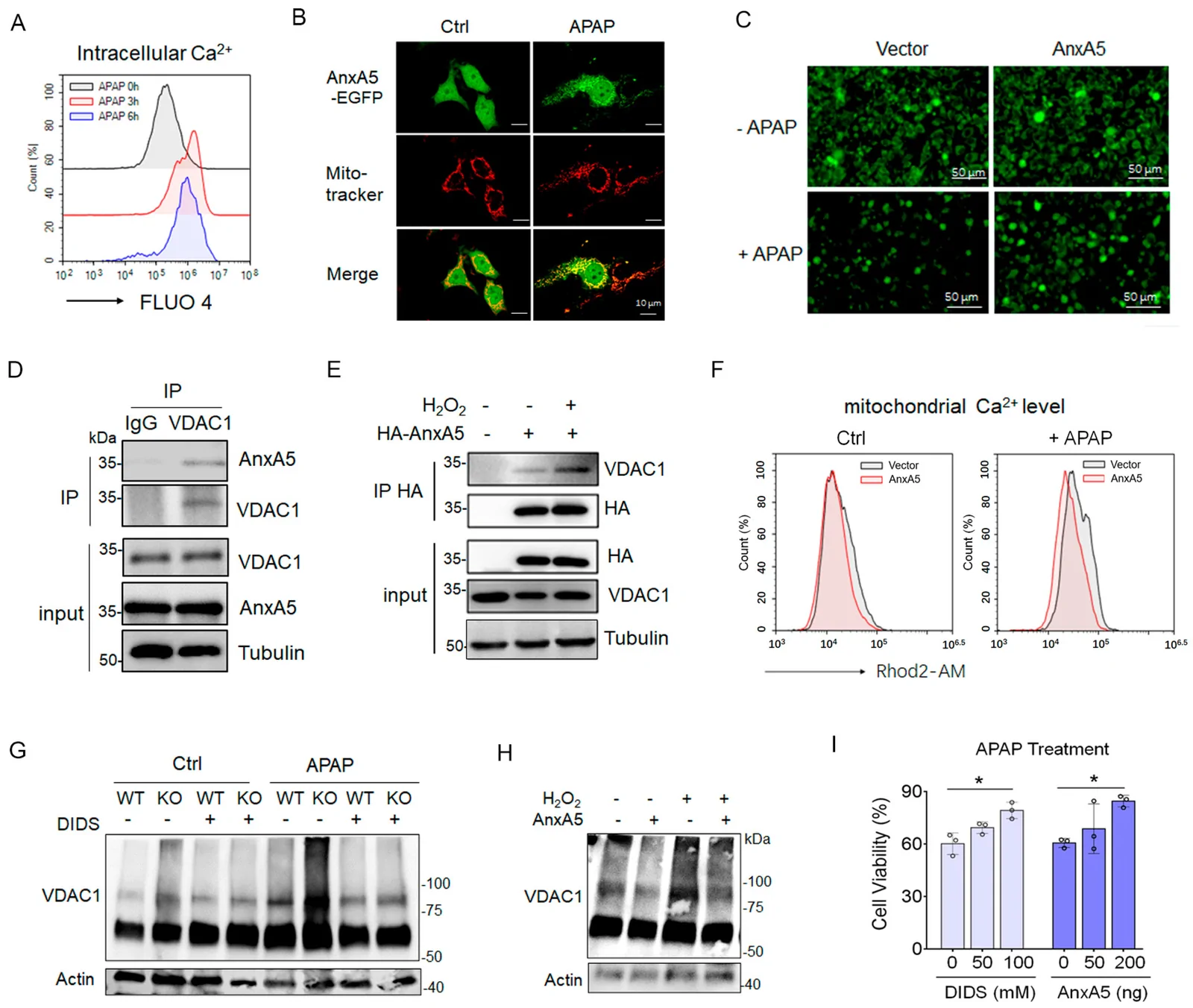
AnxA5 limits the mitochondrial mPTP opening via interacting with VDAC1. (**A**) Flow cytometric analysis of intracellular Ca^2+^ in HepG2 cells treated with 10 mM APAP for 0–3h. (**B**) Representative confocal images of AnxA5-EGFP in untreated HepG2 cells or those treated with APAP. Mitochondria labeled with mito-DsRed2 in red (scale bars, 10 µm). (**C**) Representative images of calcein-quenching assay to measure mPTP opening (scale bars, 50 µm). (**D**) Co-immunoprecipitation of AnxA5 and VDAC1 in HepG2 cells. (**E**) Cells were transfected with HA-AnxA5 and then exposed to 200 μM H_2_O_2_, after which co-immunoprecipitation assays were performed to detect the interaction between AnxA5 and VDAC1. (**F**) Flow cytometric measurement of mitochondrial Ca^2+^ levels in HepG2 cells transfected with AnxA5 or empty vector and treated with 10 mM APAP. (**G**) Representative immunoblot showing dimeric and polymeric VDAC1 levels in WT and A5KO cells treated with or without 10 mM APAP or DIDS for 6h. (**H**) After transfection, cells were treated with H_2_O_2_ for 6 h and then analyzed by immunoblotting. (**I**) HepG2 cells were subjected to either AnxA5 transfection or DIDS treatment, followed by exposure to 10 mM APAP for 24 h. Cell viability was then assessed using the CCK-8 assay. Data are presented as mean ± SD. * *p* < 0.05.

Intracellular calcium homeostasis is essential for diverse cellular processes, including ROS production and cell death regulation [19]. APAP exposure led to a marked increase in intracellular Ca^2+^ levels (Figure 3A). Given that AnxA5 is a Ca^2+^-dependent phospholipid-binding protein, we examined its subcellular localization and found that AnxA5 translocated to mitochondria upon APAP treatment (Figure 3B). APAP totoxicity primarily results from sustained oxidative stress initiated by its metabolism, which ultimately triggers the opening of the mitochondrial permeability transition pore (mPTP), a critical executioner of hepatocyte necrosis [9]. Notably, AnxA5 overexpression effectively suppressed mPTP opening (Figure 3C).

The voltage-dependent anion channel 1 (VDAC1) on the outer mitochondrial membrane has emerged as a key regulator of mPTP opening [20]. In the co-immunoprecipitation (co-IP) assays, we found that AnxA5 interacted with VDAC1 (Figure 3D), and this interaction was further enhanced by oxidative stress (Figure 3E). Given that VDAC1 serves as the main gatekeeper for mitochondrial Ca^2+^ flux [12], we next examined the functional impact of AnxA5 on mitochondrial Ca^2+^ handling. Indeed, AnxA5 overexpression significantly attenuated APAP-induced mitochondrial Ca^2+^ overload (Figure 3F).

In addition to being triggered by mitochondrial Ca^2+^ overload, mPTP opening is also promoted by VDAC1 oligomerization [21]. Under pathological conditions such as oxidative stress or calcium overload, VDAC1 self-aggregates to form oligomeric pore-like channels in the outer mitochondrial membrane, thereby disrupting mitochondrial homeostasis [22]. Consistent with this, we found that APAP treatment enhanced VDAC1 oligomerization. Notably, VDAC1 oligomerization was more pronounced in A5KO cells (Figure 3G). As expected, AnxA5 overexpression effectively suppressed VDAC1 oligomerization and alleviated cell death, similar to DIDS, a well-characterized inhibitor of VDAC1 oligomerization (Figure 3H,I). Collectively, these results suggest that ANXA5 binding to VDAC1 acts as a protective barrier that limits mPTP opening.

### 2.4. Recombinant AnxA5 Preserves Mitochondrial Integrity Against APAP Toxicity

Extracellular AnxA5 has been reported to enter cells via phosphatidylserine (PS)-mediated endocytosis [23]. To evaluate the therapeutic potential of exogenous AnxA5 in liver injury, we expressed and purified recombinant wild-type AnxA5 (A5) and its mutant A5m protein (Figure S3A), containing mutations that disrupt its calcium-dependent PS binding activity [24]. When fused with EGFP, AnxA5-EGFP was efficiently internalized into HepG2 cells, whereas A5m-EGFP failed to be taken up (Figure S3B), confirming that A5m serves as a critical negative control to exclude non-specific effects mediated by the extracellular protein.

**Figure 4:**
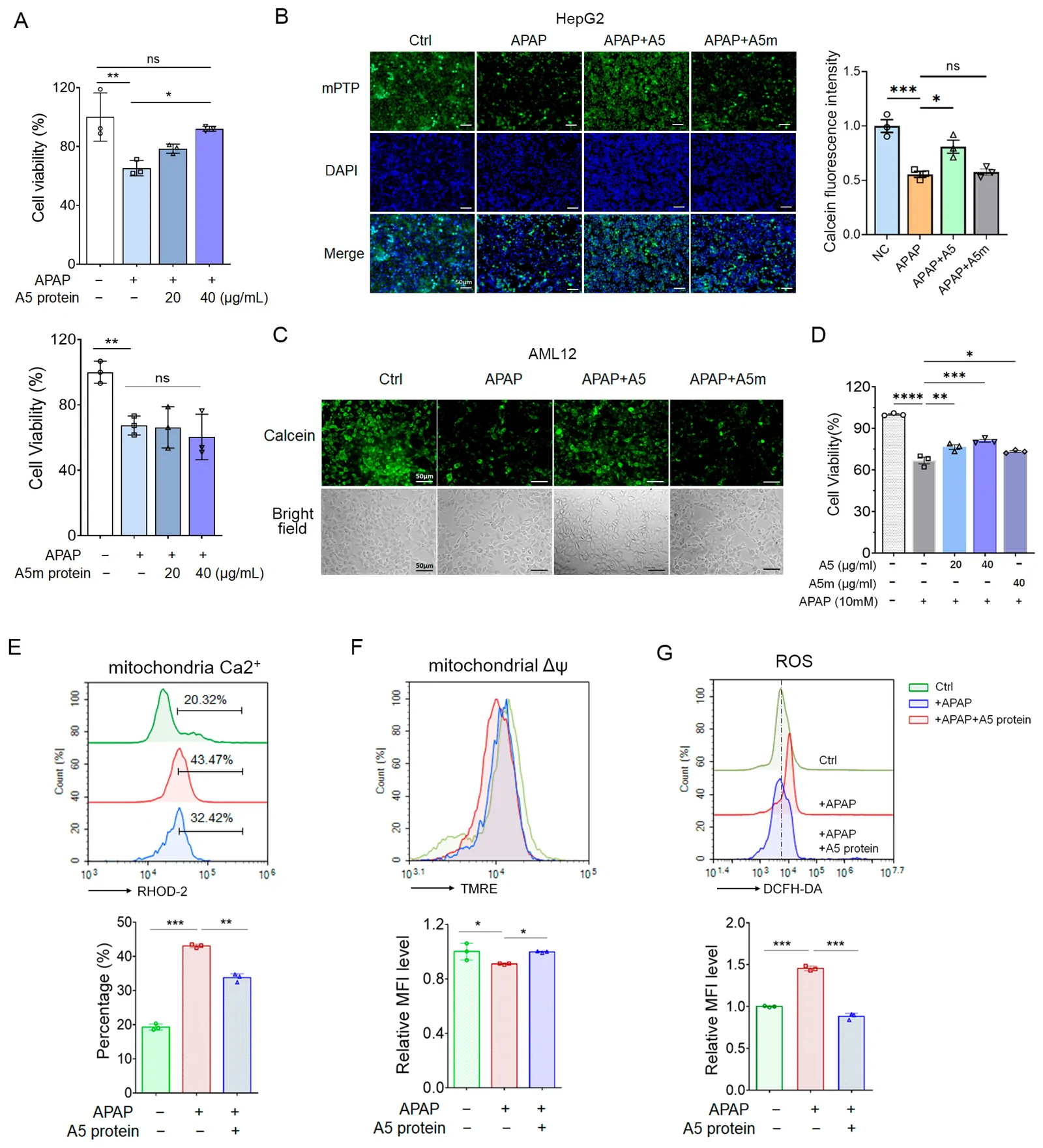
The protective effect of recombinant AnxA5 protein in response to APAP toxicity. (**A**) CCK-8 assay of HepG2 cells following 24 h co-treatment with APAP and purified AnxA5 (A5) or A5m protein. (**B**) Calcein-quenching assay to measure mPTP opening. HepG2 cells were co-incubated with 40 μg/mL A5 or A5m protein and then treated with 10mM APAP for 4h. Quantitative analysis based on calcein fluorescence intensity (scale bars: 50 µm). (**C**,**D**) AML12 cells were treated with APAP and A5 or A5m protein, calcein-quenching assay after 6 h and CCK-8 assay following 24 h co-treatment (**D**). (**E**–**G**) Flow cytometric analysis of key mitochondrial parameters in HepG2 cells with treatment of 40 μg/mL A5 protein or 10mM APAP: (**E**) Mitochondrial Ca^2+^ level (Rhod-2 AM); (**F**) Mitochondrial membrane potential (ΔΨm; TMRE); (**G**) Intracellular ROS levels (DCFH-DA). Quantitative analysis was performed based on the mean fluorescence intensity (MFI). Data presented as the mean ± SD (*n* = 3). ns (*p* > 0.05), * *p* < 0.05, ** *p* < 0.01, *** *p* < 0.001, **** *p* < 0.0001.

Consistent with its cellular internalization, treatment with recombinant A5 protein, but not the A5m mutant, significantly rescued cell viability from APAP-induced toxicity (Figure 4A). Importantly, A5 protein treatment also inhibited APAP-induced mPTP opening, thereby preserving mitochondrial integrity (Figure 4B). To further validate these findings in a more physiologically relevant model, we performed similar experiments in AML12 cells, a murine normal hepatocyte cell line. Consistently, AnxA5 protein treatment effectively suppressed mitochondrial mPTP opening and alleviated APAP-induced cytotoxicity in AML12 cells (Figure 4C,D). In line with its protective effects, A5 protein co-incubation alleviated mitochondrial calcium overload (Figure 4E), preserved the mitochondrial membrane potential (ΔΨm) (Figure 4F), and markedly reduced ROS accumulation (Figure 4G). Collectively, these findings demonstrate that exogenous supplementation of recombinant AnxA5 effectively protects mitochondrial integrity and mitigates APAP-induced toxicity.

### 2.5. Therapeutic Efficacy of Recombinant AnxA5 in a Mouse Model of APAP-Induced Liver Injury

**Figure 5:**
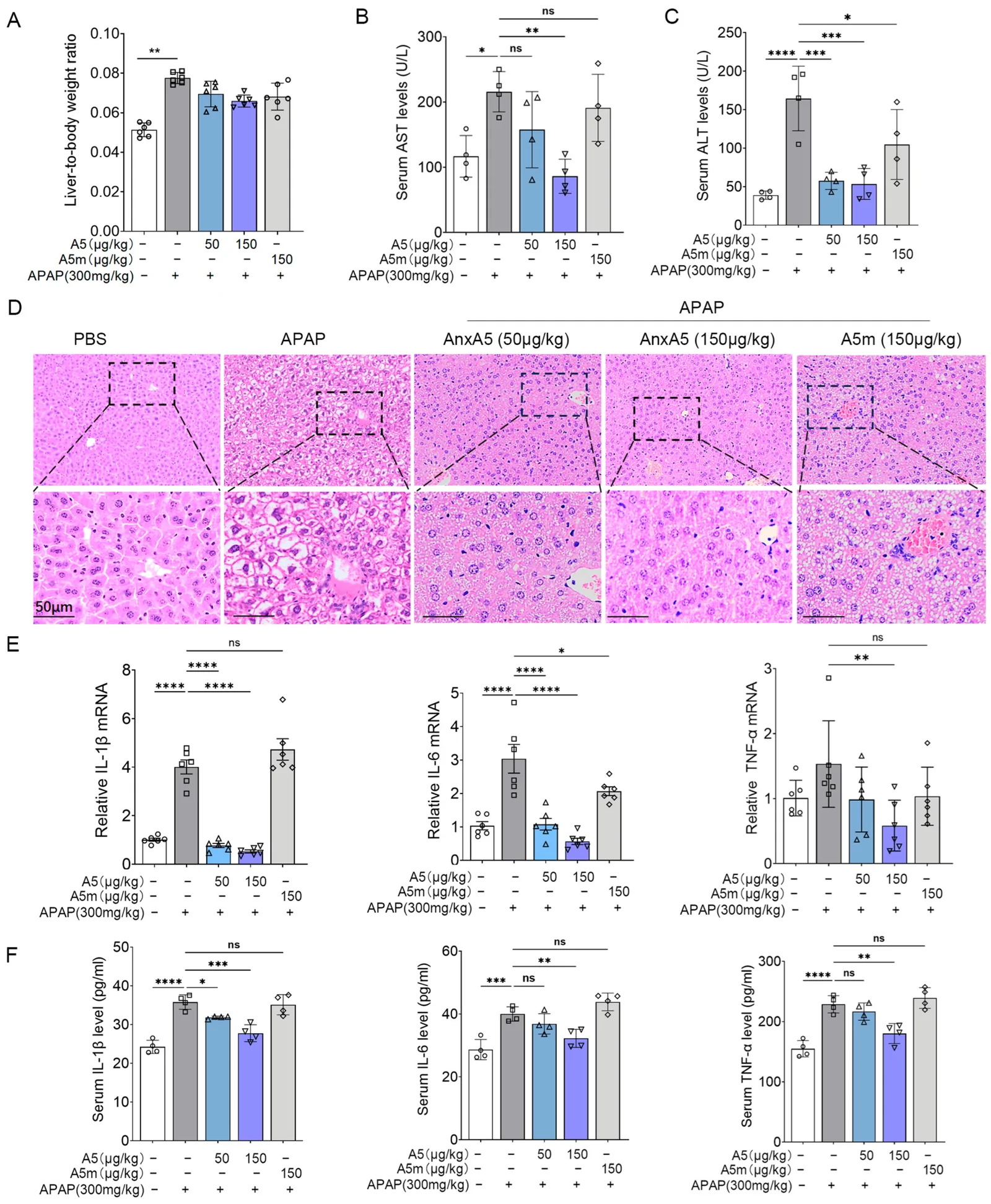
AnxA5 treatment alleviates APAP-induced hepatotoxicity in mice. The recombinant A5 or A5m protein administration via tail vein injection on APAP-induced liver injury model as described in the methods. Mice were analyzed 24 h post-administration (*n* = 4–6 per group). (**A**) Liver-to-body weight ratio. (**B**) Serum AST level. (**C**) Serum ALT level. (**D**) Representative images of H&E-stained liver sections (scale bar, 50 μm). (**E**) Hepatic mRNA expression levels of IL-1β, IL-6, and TNF-α. (**F**) Serum levels of IL-1β, IL-6, TNF-α. Data presented as the mean ± SD (*n* = 4–6). ns (*p* > 0.05), * *p* < 0.05, ** *p* < 0.01, *** *p* < 0.001, **** *p* < 0.0001.

To evaluate the therapeutic potential of recombinant AnxA5 in vivo, we established a murine model of APAP-induced acute liver injury. Mice were challenged with APAP (300 mg/kg) and subsequently treated with saline, recombinant AnxA5 (50 or 150 μg/kg), or the mutant A5m (150 μg/kg). APAP challenge significantly increased liver weight and markedly elevated serum ALT and AST levels. Administration of recombinant AnxA5, but not the mutant A5m protein, prevented APAP-induced elevation in liver weight and significantly reduced both transaminases in a dose-dependent manner (Figure 5A–C). Histological assessments of liver sections also showed only minimal histological damage in AnxA5 group, whereas the APAP and A5m groups exhibited severe vacuolation and inflammatory infiltration (Figure 5D and Figure S4). We next assessed the associated inflammatory response. In liver tissue, APAP challenge induced a marked upregulation of proinflammatory cytokines, including TNF-α, IL-6, and IL-1β. Notably, AnxA5 treatment dose-dependently suppressed the expression of these cytokines, whereas A5m had no effect (Figure 5E). Consistently, serum levels of these cytokines were significantly lower in ANXA5-treated mice (Figure 5F). These results demonstrate that AnxA5 administration effectively alleviates APAP-induced hepatic injury and the accompanying inflammatory response in vivo.

**Figure 6:**
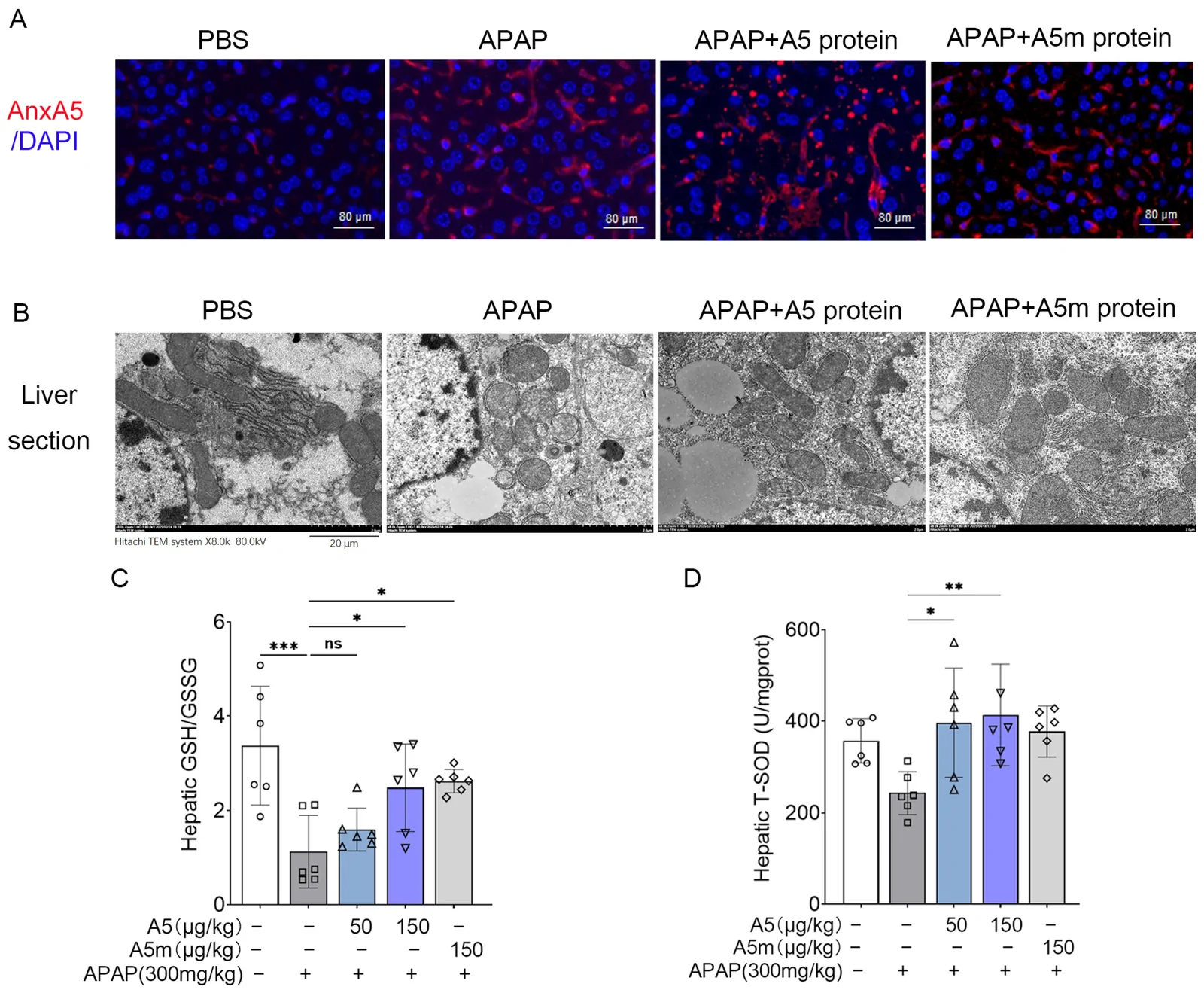
AnxA5 preserves mitochondrial integrity and enhances antioxidant defenses in vivo. (**A**) Representative immunofluorescence of AnxA5 (red) in liver section from mice in the indicated groups (scale bar, 80 μm). (**B**) Representative electron microscopy images of hepatic mitochondria (scale bar, 20 μm). (**C**) GSH/GSSG ratio in liver tissue (*n* = 6 each group). (**D**) Total SOD activity in liver tissue (*n* = 6 each group). Data are presented as the mean ± SD. ns (*p* > 0.05), * *p* < 0.05, ** *p* < 0.01, *** *p* < 0.001.

Given that APAP-induced hepatotoxicity is primarily driven by mitochondrial dysfunction, we further examined the impact of AnxA5 on mitochondrial integrity. Immunofluorescence of liver sections showed that endogenous AnxA5 protein levels were elevated upon APAP challenge, and therapeutic administration of recombinant AnxA5 significantly augmented the overall AnxA5 levels in hepatic tissue (Figure 6A). Consistent with these enhanced AnxA5 levels, electron microscopy demonstrated that the APAP-induced mitochondrial swelling and cristae disruption were alleviated by AnxA5 treatment, and mitochondria in the AnxA5 group displayed nearly normal morphology (Figure 6B). The protective effect of AnxA5 on mitochondria led to restoration of GSH levels in the liver, indicating attenuation of APAP-induced oxidative stress (Figure 6C). Moreover, we observed a significant increase in total SOD activity following AnxA5 treatment (Figure 6D), which is likely to have been due to enhanced mitochondrial SOD2 function, further supporting the improved mitochondrial integrity. Taken together, these in vivo data demonstrate that AnxA5 confers resistance to APAP-induced liver injury by preserving mitochondrial functional integrity.

## 3. Discussion

Mitochondrial dysfunction is recognized as a driving force in the pathogenesis and progression of liver diseases. Impaired mitochondrial integrity leads to sustained oxidative stress, a critical causative factor of hepatocyte death and liver injury. In the context of APAP overdose, the accumulation of the toxic metabolite NAPQI triggers mitochondrial oxidative stress, ultimately culminating in acute liver failure [25,26]. In this study, we identify AnxA5 as an endogenous regulator of mitochondrial homeostasis, which is significantly upregulated in both human and murine livers under APAP-induced stress. Through its interaction with VDAC1, AnxA5 limits pathological mitochondrial calcium overload and prevents mPTP opening, thereby attenuating APAP-induced oxidative stress injury. These findings provide proof of concept for the protective role of AnxA5 and highlight AnxA5 as a potential lead for further exploration as therapy against APAP-induced liver injury.

Upon Ca^2+^ elevation, AnxA5 is known to translocate from the cytosol to various subcellular compartments, including the plasma membrane, endoplasmic reticulum and mitochondria [11,12]. Consistently, APAP treatment resulted in an increased intracellular Ca^2+^ levels, and we observed a marked mitochondrial translocation of AnxA5. Mechanistically, co-IP assays showed the enhanced interaction of AnxA5 with VDAC1 under oxidative stress. Molecular dynamics simulations revealed that the AnxA5-VDAC1 complex is thermodynamically stable, as evidenced by a negative binding free energy (−ΔG) (Figure S5). Functionally, the association of AnxA5 with VDAC1 exerted multiple protective effects on mitochondria, including alleviation of mitochondrial Ca^2+^ overload, inhibition of VDAC1 oligomerization, and suppression of mPTP opening (Figure 3). Upon APAP induction, VDAC1 underwent oligomerization, which promoted mPTP opening and led to mitochondrial swelling and rupture. Inhibition of VDAC1 oligomerization with DIDS reduced cell death, as well as AnxA5 overexpression. Our study links AnxA5 to the regulation of VDAC1 oligomerization, a process crucial for maintaining mitochondrial integrity. These results posit a crucial role for AnxA5 in mitochondrial quality control and cell survival under stress conditions, which might be involved in mitochondrial regulatory mechanisms.

Although previous studies have reported that AnxA5 controls VDAC1-dependent mitochondrial Ca^2+^ homeostasis and influences cellular susceptibility to apoptosis [12,13,14], our study places greater emphasis on the protective role of AnxA5 in preserving mitochondrial integrity, and more importantly, on its translational potential as a therapeutic agent. We provide in vitro evidence that exogenously supplied recombinant AnxA5 is efficiently internalized by cells and exerts direct mitochondrial protection. Furthermore, in vivo experiments in a mouse model of APAP-induced liver injury demonstrated that administration of recombinant AnxA5 significantly ameliorated liver damage, reduced oxidative stress, and preserved mitochondrial ultrastructure. One limitation of the present study is the relatively modest sample size for the mouse experiments. Although statistically significant inter-group differences were detected under the current sample numbers, future investigations using larger animal cohorts (e.g., *n* = 8 mice per group) would help to further validate and consolidate our observations. Collectively, our data demonstrate that AnxA5 confers therapeutic protection against APAP-induced hepatotoxicity. Given that both ferroptosis and cuproptosis are tightly associated with mitochondrial oxidative stress, AnxA5 may exert analogous protective effects and be implicated in other diseases linked to these two forms of cell death.

From the perspective of protecting mitochondria-centric pathways, our findings highlight the capacity of AnxA5 to preserve mitochondrial function for drug-induced liver injury. Nevertheless, several critical preclinical characterizations, including pharmacokinetic profiles, tissue distribution, persistence of biological activity, rationale for dosage selection, and therapeutic window assessment, remain to be determined. Further studies are therefore required to comprehensively evaluate its translational potential for clinical application. Of note, the translational potential of AnxA5 is further supported by its established safety profile in humans. Recombinant human AnxA5 (SY-005; Suzhou Yabao Pharmaceutical R&D Co., Ltd., Suzhou, China) has been evaluated in a phase I clinical study in healthy subjects (NCT04217629) and is currently being investigated in a phase IIa trial for sepsis (NCT04898322). Our own preclinical safety assessments also demonstrated that high-dose administration of recombinant AnxA5 (700 μg/kg) did not induce toxicity in healthy mice, as evidenced by unaltered body weight and the absence of histopathological alterations in the liver and kidney [24]. Moreover, radioisotope-labeled recombinant human AnxA5 is already used clinically as a diagnostic imaging agent for detecting cell death in tumors [27,28]. In addition, AnxA5 has shown therapeutic promise in various other diseases, including vascular thrombosis, autoimmune disorders, lung injury, and cancer [29,30,31]. In conclusion, we propose that recombinant AnxA5 represents a viable therapeutic strategy to mitigate mitochondrial dysfunction in DILI, warranting further clinical development.

## 4. Materials and Methods

### 4.1. Reagents

Acetaminophen (APAP, purity > 99.98%) was purchased from MedChem Express (MCE, Monmouth Junction, NJ, USA). Commercial assay kits for alanine aminotransferase (ALT) and aspartate aminotransferase (AST) were obtained from Maike Biotechnology (Chengdu, China) and Sevier Biotechnology (Wuhan, China), respectively. Fluorescent probes and kits for reactive oxygen species (ROS), mitochondrial membrane potential (TMRE), and mitochondrial Ca^2+^ (Rhod-2 AM) were purchased from Keygen Biotech (Nanjing, China), Beyotime Biotechnology (Shanghai, China), and Yeasen Biotech (Shanghai, China), respectively. Recombinant human annexin A5 (A5) and its mutant (A5m) were expressed and purified in our laboratory (Prof. Zichun Hua’s lab, Nanjing University).

### 4.2. Animal Models

All animal experiments were according to ethical guidelines and were approved by the Institutional Animal Care and Use Committee (IACUC) of Nanjing University (Approval No: IACUC-D2303092; Approved on 9 March 2023). Male C57BL/6 mice (6–8 weeks old) were purchased from Cavens Laboratory Animal Co., Ltd. (Changzhou, China) and housed under specific pathogen-free conditions with a 12-h light/dark cycle. Mice were fasted overnight and then randomly divided into five groups (*n* = 6 per group), injected with PBS (vehicle group), or 300 mg/kg APAP (APAP group), or received i.p. APAP (300 mg/kg) followed by intravenous administration of recombinant AnxA5 (50 or 150 μg/kg) or its mutant A5m (150 μg/kg). Mice were euthanized at 24 h or 48 h post-APAP injection. Blood and liver tissues were collected for subsequent analyses.

### 4.3. Cell Culture and Treatments

The human hepatocellular carcinoma cell line HepG2 (ATCC^®^ HB-8065™) and the non-transformed mouse hepatocyte cell line AML12 (ATCC^®^ CRL-2254™) were used in this study. Cells were cultured in Dulbecco’s Modified Eagle Medium (DMEM; Gibco, Waltham, MA, USA) supplemented with 10% fetal bovine serum (FBS; HyClone, Logan, UT, USA) and 1% penicillin–streptomycin (Sangon Biotech, Shanghai, China) at 37 °C in a humidified incubator containing 5% CO_2_. For APAP cytotoxicity assays, cells were treated with indicated concentrations of APAP (0–15 mM) for 12–24 h. For rescue experiments, cells were co-incubated with 10mM APAP and purified recombinant AnxA5 (A5) or its mutant (A5m) protein (0–40 μg/mL) for 24 h.

### 4.4. Cell Viability Assay

Cell viability was determined using a Cell Counting Kit-8 (CCK-8; Novozyme, Nanjing, China). Following APAP treatment, 10 μL of CCK-8 reagent was added to each well of a 96-well plate and incubated for 2 h according to the manufacturer’s instructions. Absorbance was measured at 450 nm with a reference wavelength of 650 nm using a microplate reader (BioTek, Winooski, VT, USA). All experiments were performed in at least three independent replicates.

### 4.5. Bioinformatic Analysis

The gene expression dataset GSE120652 was downloaded from the Gene Expression Omnibus (GEO). This dataset contains microarray profiles from liver tissues of three acetaminophen-induced liver injury (AILI) patients and three healthy controls. Differential expression genes (DEGs) were defined with log2 fold change (|log2FC|) > 0.5 and an *p*-value < 0.05. Visualization of the DEGs in volcano plot and expression patterns of AnxA5 were generated using the ggplot2 (version 4.0.3), ggpubr (version 0.6.0), and pheatmap (version 1.0.13) packages, respectively.

### 4.6. RNA-Seq Analysis

Total RNAs from A5KO and WT HepG2 cells were extracted using a TRIzol reagent kit (Invitrogen, Waltham, MA, USA). Multiplex RNA-Seq analysis was outsourced to Novogene Co., Ltd. (Beijing, China). The raw reads were trimmed and quality controlled by Fastp software (v0.23.4) with default parameters. Differential gene expression analysis was performed using DESeq2 software (v1.42.0), with the screening criteria set at an adjusted *p*-value < 0.05 and |log2FC| ≥ 1. Bioinformatic analysis was conducted using GSEA analysis (http://www.broadinstitute.org/gsea/index.jsp (accessed on 27 September 2025). GO and KEGG datasets were used independently for GSEA.

### 4.7. Reverse Transcription-Quantitative Real-Time PCR (RT-qPCR)

Total RNA was prepared using the TRIzol Reagent (Invitrogen, Waltham, MA, USA). A total of 1 µg of RNA was reverse-transcribed using the TransScript^®^ All-in-One First-Strand cDNA Synthesis SuperMix for qPCR (AT341, TransGen Biotech, Beijing, China). Real-time PCR was performed with ChamQ SYBR qPCR Master Mix (Q311, Vazyme, Nanjing, China) on a StepOne Plus real-time PCR system (Applied Biosystems, Waltham, MA, USA). Quantitation of all target gene expression was normalized to the control gene β-actin for mouse or human genes. The primers used in this paper are listed in Supplementary Table S1.

### 4.8. Immunoprecipitation and Immunoblot Analysis

Cells were lysed with RIPA buffer containing 1% protease inhibitor PMSF for 40 min at 4 °C. Lysates were measured and equal amounts were used for immunoprecipitation. After the antibody was added and slowly rotated overnight at 4 °C, protein A/G magnetic beads were used to precipitate the immune complex for 1 h at 4 °C. Then, the immunocomplexes were washed with PBST (1% TritonX-100), and eluted with sample buffer containing 1% SDS for 5 min at 95 °C. The immunoprecipitated proteins were separated by SDS-PAGE. Immunoblot analysis was performed with anti-AnxA5 (Cat#11060, Proteintech, Rosemont, IL, USA) or anti-VDAC1 (Cat#66345, Proteintech, Rosemont, IL, USA) antibody and secondary anti-mouse or anti-rabbit antibodies conjugated to HRP (Servicebio, Wuhan, China). Visualization was achieved with chemiluminescence.

### 4.9. Measurement of ROS, Mitochondrial Ca^2+^ and ΔΨm

After APAP treatment or transfection, cells were harvested, washed with PBS, and stained with specific fluorescent probes according to the manufacturers’ protocols: DCFH-DA for ROS detection, TMRE for mitochondrial membrane potential (ΔΨm), and Rhod-2 AM for mitochondrial Ca^2+^ levels. Following incubation and washing, stained cells were resuspended in assay buffer and immediately analyzed using a NovoCyte flow cytometer (Agilent Technologies, Santa Clara, CA, USA). Data were processed using NovoExpress software (v2.2.0, Agilent Technologies).

### 4.10. The mPTP Opening Assay

A Mitochondrial Permeability Transition Pore Assay Kit (Abbkine, Wuhan, China) was utilized. Briefly, HepG2 cells were seeded in 12-well plates and treated with 10 mM APAP for 24h. Then, cells were harvested and incubated with 1 µM Calcein-AM and 10 µM CoCl_2_ in serum-free culture medium for 40 min at 37 °C in the dark. Subsequently, the medium was replaced with fresh culture medium, and the cells were incubated again in the dark at 37 °C for an additional 30 min. After washing with PBS, the retained mitochondrial calcein fluorescence was observed and imaged using a Zeiss inverted fluorescence microscope (Zeiss, Oberkochen, Germany).

### 4.11. Subcellular Localization

HepG2 cells were seeded onto sterile glass coverslips in 12-well plates and transfected with the AnxA5-EGFP expression plasmid and the mito-DsRed2 plasmid. After 24 h, cells were treated with APAP (or vehicle control) for a further 24 h. Following treatment, the culture medium was removed, and cells were washed twice with PBS. The coverslips were then mounted on glass slides. High-resolution images of AnxA5-EGFP (green) and mito-DsRed2 (red) were captured using an Olympus FV4000 laser scanning confocal microscope (Olympus Corporation, Tokyo, Japan).

### 4.12. VDAC1 Oligomerization

After 6 h of APAP or DIDS treatment, HepG2 cells were washed with precooled PBS and collected together in PBS with 0.5 mM EGS (Thermo Fisher Scientific, Waltham, MA, USA), and incubated at 30 °C for 30 min for intercellular VDAC1 crosslinking. The crosslink reaction was quenched by the addition of 5 mM Tris-Cl (pH 7.5), and the cells were lysed for subsequent immunoblotting of VDAC1 oligomerization using VDAC1 antibody.

### 4.13. Assessment of Liver Injury

The liver index was calculated as liver wet weight/body weight. Then, liver tissues were fixed in 4% paraformaldehyde, paraffin-embedded, sectioned, and stained with hematoxylin and eosin (H&E). Liver-injury severity was scored histologically using the Suzuki-type scoring system under a SLIDEVIEW VS200 light microscope (Evident-Olympus, Tokyo, Japan). The scoring parameters included the extent of hepatocellular necrosis, vacuolization, and inflammatory infiltration.

For transmission electron microscopy, fresh liver tissue was fixed in 2.5% glutaraldehyde, processed, and ultrathin sections were stained with uranyl acetate and lead citrate. Mitochondrial and cellular ultrastructure were examined using a Hitachi HT7800/7700 TEM (Hitachi High-Tech Corporation, Tokyo, Japan).

For the biochemical assay, the levels of alanine aminotransferase (ALT) and aspartate aminotransferase (AST) in the serum were measured using a commercially available assay kit following the manufacturer’s instructions.

### 4.14. Measurement of Oxidative Stress and Inflammatory Markers

The GSH and GSSG Assay Kit (Cat. No. S0053) and Total-SOD activity assay kit (Cat. No. S0101S) and were obtained from Beyotime Biotechnology (Shanghai, China). For the assessment of hepatic antioxidant status, liver homogenates were prepared, and supernatants were used to determine superoxide dismutase (SOD) activity and the reduced/oxidized glutathione (GSH/GSSG) ratio, following the kit protocols.

For analysis of serum cytokines, the levels of interleukin-1β (IL-1β), interleukin-6 (IL-6), and tumor necrosis factor-α (TNF-α) were measured using commercially available ELISA detection kits (YouMeng Biotechnology, Nanjing, China).

### 4.15. Statistical Analysis

All data are presented as mean ± SD. Statistical significance was determined by one-way or two-way ANOVA followed by an appropriate post hoc test, using GraphPad Prism software (version 9.0.0). A *p*-value < 0.05 was considered statistically significant.

## Data Availability

All data and plasmids supporting the findings of this study are available from the corresponding authors on reasonable request.

## Supplementary Materials

The following supporting information can be downloaded at: https://www.mdpi.com/article/10.3390/ijms27177771/s1.
