# Supplementary material for: Annexin A5 Maintains Mitochondrial Integrity by Inhibiting mPTP Opening to Protect Against Acetaminophen-Induced Acute Liver Injury
Source: Int J Mol Sci. 2026 Aug 30;27(17):7771. doi: 10.3390/ijms27177771 (PMC13566027; doi:10.3390/ijms27177771)
Supplement: Supplementary file 1 [file ijms-27-07771-s001.zip › ijms-4459047-supplementary.pdf]

Supplementary Material

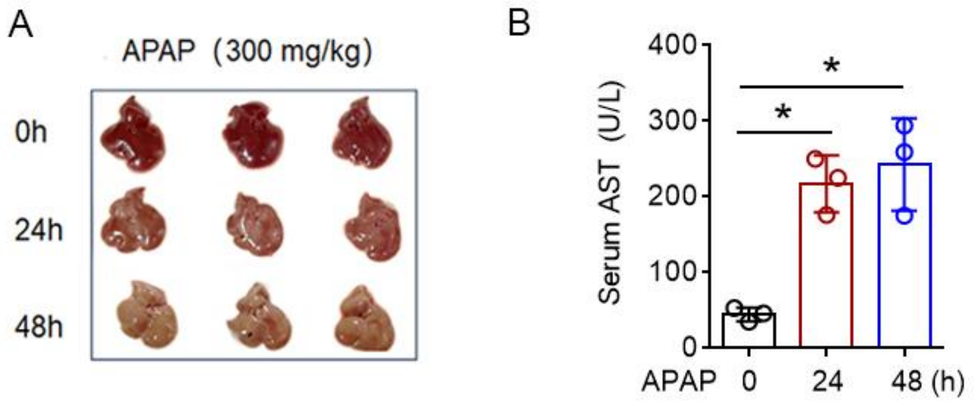

Figure S1. The effects of APAP on the liver of mice**.**

(A) Representative images of the livers from mice treated with APAP (300 mg/kg) for the indicated durations. (B) Serum AST level in APAP-treated mice.

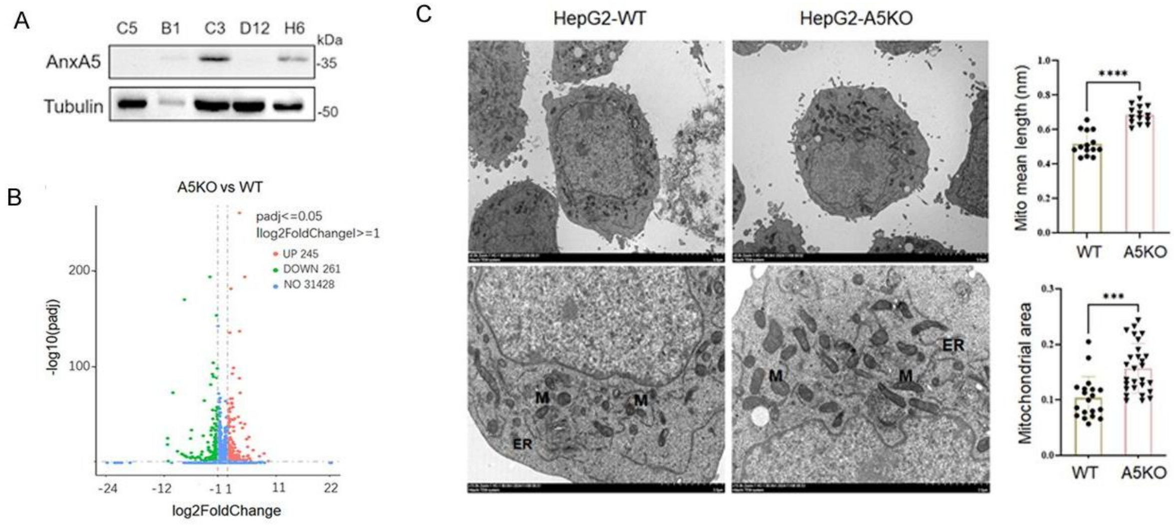

Figure S2. AnxA5 deficiency causes mitochondrial morphological alterations.

(A) The validation of AnxA5 knockout cells by western blot .C5 and D12 are AnxA5 knockout (A5KO) cells. (B) Volcano plot of the differentially genes between WT and A5KO cells. (C) Representative electron micrographs of WT and A5KO cells. Mitochondrial size based on length and area measurements.

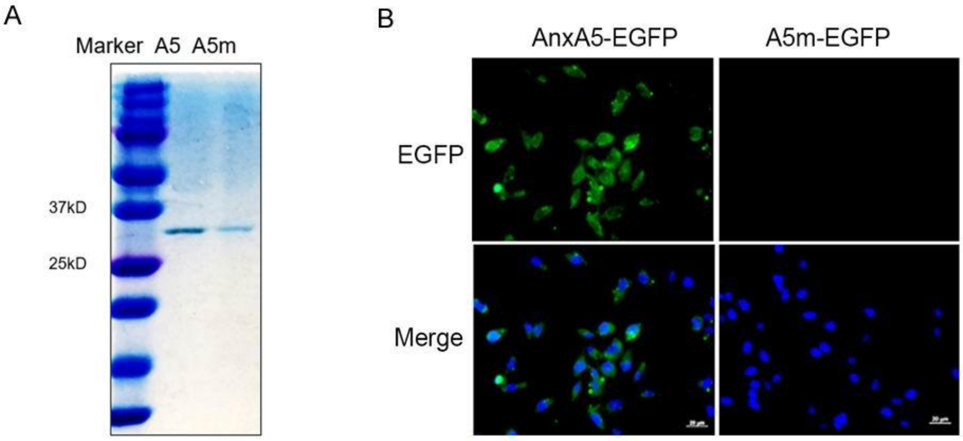

Figure S3. The complementation analysis of recombinant A5 and A5m proteins

(A) The purified recombinant proteins of A5 and A5m. (B) used for treatment of APAP model mice. (B) Cellular uptake of recombinant A5-EGFP protein was assessed by fluorescence microscopy after a 3-hour co-incubation with HepG2 cells. A5m-EGFP as a negative control.

Histological criteria for the assessment of liver damage after hepatectomy

Numerical assessment	Congestion	Vacuolation	Necrosis
0	None	None	None
1	Minimal	Minimal	Single cell
2	Mild	Mild	<30%
3	Moderate	Moderate	<60%
4	Severe	Severe	>60%

Suzuki's criteria were modified from Suzuki *et al*, 1993

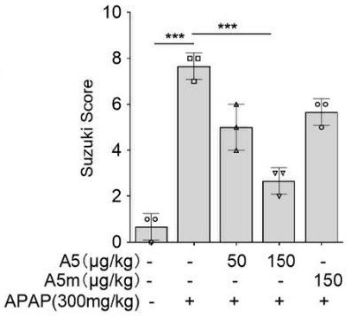

Figure S4. Suzuki scoring of hepatic injury in experimental groups.

The standards of Suzuki score for liver injury and Suzuki score of H&E-stained liver sections from each experimental group (*n* = 3 per group; mean ± SD). ***p < 0.001 vs. APAP group.

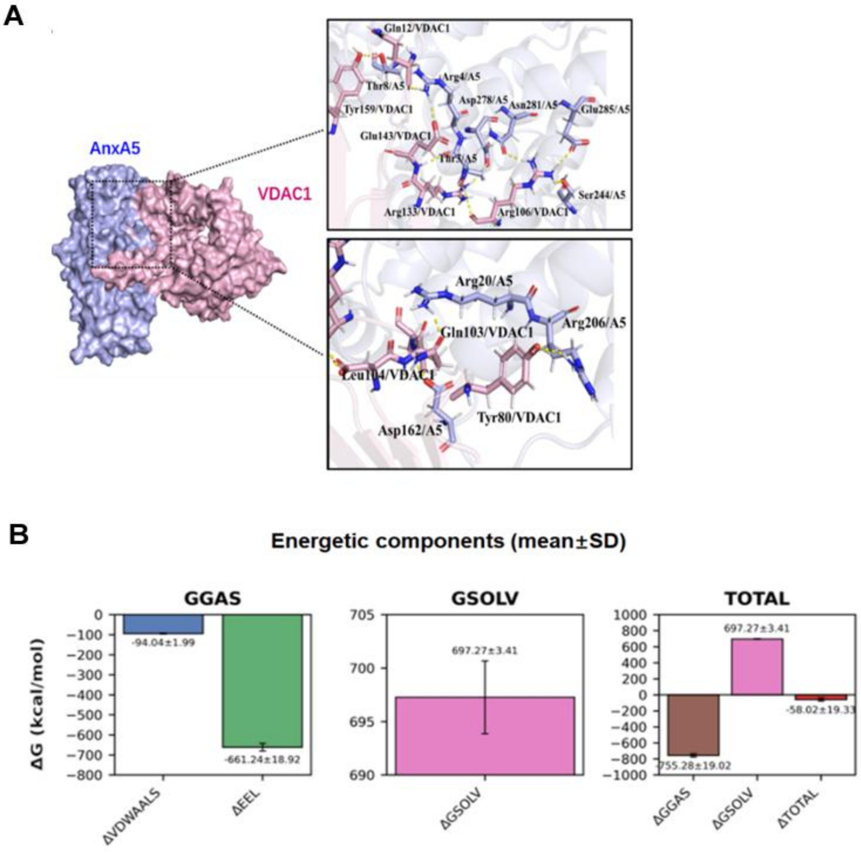

Figure S5. Computational analyses of the interaction between AnxA5 and VDAC1**.** **(A)** Molecular docking model of the ANXA5-VDAC1 complex, highlighting There is a stable hydrogen-bonding network involving residues Thr3, Arg4, and Thr8 in the N-terminal region of AnxA5, as well as Ser244, Asp278, and Asn281 in its domain IV, which form hydrogen bonds with specific residues in VDAC1; key hydrogen bonds (in yellow). **(B)** The MMPBSA binding free-energy analysis reveals a total ΔG ≈ -58.02 ± 19.33 kcal.mol^{-1}, indicating substantial thermodynamic stabilization upon complex formation. Energetic components derived from the MM/PBSA analysis, including van der Waals (ΔVDWAALS), electrostatic (ΔEEL), polar solvation (ΔEPB), and nonpolar solvation (ΔENPOLAR) terms.

Table S1 Primer sequences for qPCR

Gene	Primer sequence
human β-actin	F: 5-GGACTTCGAGCAAGAGATGG<br>R:5-AGCACTGTGTTGGCGTACAG
human Anxa5	F: 5-CTTGGGCACAGATGAGGAGAGCA<br>R:5- AAGCCGAGAGGGTTTCATCAGAGC
mouse β-actin	F: 5-GGCTGTATTCCCCTCCATCG<br>R: 5-CCAGTTGGTAACAATGCCATGT
mouse Anxa5	F: 5-TGAGGACAGCATCCTGAACCTG<br>R: 5-CAGACTTCAGGTCATCCACAAGG
mouse IL-1β	F: 5-GAAATGCCACCTTTTGACAGTG<br>R: 5-TGGATGCTCTCATCAGGACAG
mouse TNF-α	F: 5-ACCACGCTCTTCTGTCTACT<br>R: 5-AGGAGGTTGACTTTCTCCTG
mouse IL-6	F: 5-CCAAGAGGTGAGTGCTTCCC<br>R: 5-CTGTTGTTCAGACTCTCTCCCT
